# The effects of short‐chain fatty acids on the transcription and secretion of apolipoprotein A‐I in human hepatocytes in vitro

**DOI:** 10.1002/jcb.28982

**Published:** 2019-05-20

**Authors:** Jehad Z. Tayyeb, Herman E. Popeijus, Ronald P. Mensink, Maurice C. J. M. Konings, Kim H. R. Mulders, Jogchum Plat

**Affiliations:** ^1^ Department of Nutrition and Movement Sciences, NUTRIM School for Nutrition and Translational Research in Metabolism Maastricht University Maastricht The Netherlands

**Keywords:** antibiotics, ApoA‐I, BET, PPARα, SCFA, transcription

## Abstract

**Background:**

Apolipoprotein‐I (ApoA‐I), the major component of high‐density lipoprotein (HDL) particles, mediates cholesterol efflux by which it facilitates the removal of excess cholesterol from peripheral tissues. Therefore, elevating ApoA‐I production leading to the production of new pre‐β‐HDL particles is thought to be beneficial in the prevention of cardiovascular diseases. Recently, we observed that amoxicillin treatment led to decreased HDL concentrations in healthy human volunteers. We questioned whether this antibiotic effect was directly or indirectly, via changed short‐chain fatty acids (SCFA) concentrations through an altered gut microflora. Therefore, we here evaluated the effects of amoxicillin and various SCFA on hepatic ApoA‐I expression, secretion, and the putative underlying pathways.

**Methods and Results:**

Human hepatocytes (HepG2) were exposed to increasing dose of amoxicillin or SCFA for 48  hours. ApoA‐I messenger RNA (mRNA) transcription and secreted protein were analyzed using quantitative polymerase chain reaction and enzyme‐linked immunosorbent assay, respectively. To study underlying mechanisms, changes in mRNA expression of KEAP1, CPT1, and PPARα, as well as a PPARα transactivation assay, were analyzed. Amoxicillin dose‐dependently decreased ApoA‐I mRNA transcription as well as ApoA‐I protein secretion. SCFA treatment resulted in a dose‐dependent stimulation of ApoA‐I mRNA transcription, however, the ApoA‐I protein secretion was decreased. Furthermore, SCFA treatment increased PPARα transactivation, PPARα and CPT1 mRNA transcription, whereas KEAP1 mRNA transcription was decreased.

**Conclusion:**

Direct treatment of HepG2 cells with amoxicillin has either direct effects on lowering ApoA‐I transcription and secretion or indirect effects via modified SCFA concentrations because SCFA were found to stimulate hepatic ApoA‐I expression. Furthermore, BET inhibition and PPARα activation were identified as possible mechanisms behind the observed effects on ApoA‐I transcription.

## Graphical abstract

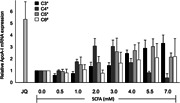

## 1 INTRODUCTION

Several studies have suggested that antibiotics affect lipid and lipoprotein metabolism.^1^, ^2^ In fact, in a recent placebo‐controlled trial we observed that amoxicillin treatment for 7 days lowered high‐density lipoprotein (HDL) cholesterol concentrations in healthy volunteers.^2^ Whether this decrease is harmful as related to cardiovascular diseases (CVDs)‐risk, is questionable because HDL functionality may be more important in this respect than HDL cholesterol concentrations.^3^ These functional, antiatherogenic effects have been mainly attributed to its most predominant structural protein particle, apolipoprotein‐I (ApoA‐I), which is produced by the small intestine and the liver.^4^ Amongst others, ApoA‐I mediates cholesterol efflux facilitating the removal of excess cholesterol from peripheral tissues and the uptake by the liver for excretion into bile.^5^ Since ApoA‐I is one of the most important HDL proteins in terms of functionality, a better understanding of ApoA‐I transcription and production is warranted. Therefore, we explored in vitro the potential antibiotic‐related effects on ApoA‐I transcription and synthesis. Effects of antibiotics on *de novo* ApoA‐I expression can be a direct effect of the antibiotic on gene transcription or an indirect effect by affecting microbiota composition. Effects of antibiotics on ApoA‐I transcription have to the best of our knowledge never been studied. Regarding indirect effects, Reijnders et al^1^ have recently shown that the decreased bacterial diversity after the intake of vancomycin resulted in reduced circulating short‐chain fatty acids (SCFA) concentrations. These SCFA are produced in the colon by the fermentation of dietary fibers such as resistant starches and nonstarch polysaccharides.^6^ SCFA have been linked to multiple beneficial health effects including a reduced risk of inflammatory diseases, gastrointestinal disorders, cancer, and CVD.^7^ Furthermore, it was recently shown that dietary SCFA increase HDL cholesterol concentrations in hamsters.^8^ We, therefore, hypothesized that antibiotics directly, or indirectly via reduced SCFA concentrations, may relate to the changes in ApoA‐I transcription, which eventually led to decreased HDL cholesterol concentrations.

Besides evaluating the effects of SCFA on hepatic ApoA‐I expression, an additional question is how these potential effects are regulated at a molecular level. Previous studies reported that both bromodomain and extra‐terminal domain (BET) inhibition and PPARα activation had a major effect on ApoA‐I transcription.^9^, ^10^ For example, BET protein inhibitors such as JQ1(+) increased ApoA‐I expression and protein secretion in human hepatocellular liver carcinoma (HepG2) cells.^11^, ^12^ BET inhibitors can bind to BET proteins such as BRD4, a general transcriptional regulator, which can regulate transcription of target genes such as KEAP1.^13^, ^14^ PPARα is a nuclear receptor that forms a heterodimer with the retinoid X receptor, which then binds to specific response elements (PPREs) within promoter regions of target genes such as PPARα itself, CPT1,^15^ and ApoA‐I.^16^ Various dietary components such as long‐chain fatty acids have been recognized as natural ligands for PPARα,^17^ but there are indications that SCFA may have similar effects.^18^, ^19^ Therefore, except for effects on changes in ApoA‐I messenger RNA (mRNA), we also evaluated changes in KEAP1, CPT1, and PPARα mRNA expressions during exposure of HepG2 cells to different SCFA to examine potential underlying pathways.

## 2 MATERIAL AND METHODS

### 2.1 Materials

HepG2 cells were kindly provided by Sten Braesch‐Andersen (Mabtech, Nacka Strand, Sweden). Cell culture flasks and plates were obtained from Corning (Cambridge, MA). Minimum essential medium (MEM), sodium pyruvate, nonessential amino acids (NEAA), and penicillin and streptomycin were all obtained from Thermo Fisher Scientific (Bleiswijk, The Netherlands). Fetal bovine serum (FBS) was purchased from PAA (Toronto, Canada). Amoxicillin was obtained from Sigma (Uithoorn, The Netherlands). Propionic acid (C3), butyric acid (C4), valeric acid (C5), and hexanoic acid (C6) were purchased from Sigma. The BET inhibitor JQ1(+), was purchased from Bio‐techne ‐ R&D (Minneapolis, MN). Thapsigargin (Taps), a endoplasmic reticulum (ER)‐stress inducer, was purchased from Sigma. Dimethyl sulfoxide (DMSO) and TRI reagent were obtained from Sigma.

### 2.2 Cell culture and SCFA treatment

HepG2 cells were cultured at 37°C in a humidified atmosphere of 5% carbon dioxide (CO_2_) in MEM containing 10% heat‐inactivated FBS, 1% sodium pyruvate, 1% NEAA, and 1% of penicillin‐streptomycin mixture. In the amoxicillin experiments, cells were cultured without penicillin‐streptomycin mixture. For all experiments, cells were seeded in a 24‐well plate at a density of 200 000 cells per well. Cell viability was daily inspected by microscope and when cells reached a density of 80%‐90%, they were incubated for 48 hours in the medium (MEM without FBS) plus a concentration range of 0 to 7 mM SCFA (C3, C4, C5, or C6) or amoxicillin (0‐200 µg/mL) or 3 µM JQ1(+). The positive control JQ1(+) was included—separate from the SCFA—in all experiments to ensure the cells were responsive and capable to produce sufficient amounts of ApoA‐I mRNA. All SCFA as well as JQ1(+) were dissolved in DMSO, (cell culture tested) and effects were expressed relative to those of the carrier control (DMSO only). Final DMSO concentration was always 0.2%. As amoxicillin was dissolved in water, the effects of amoxicillin were compared with those of a water control. In previous study,^20^ it was shown that after oral administration of 500 mg of amoxicillin (taken three times a day), serum peak levels were between 6.0 and 15.3 μg/mL and after intravenous administration of 500 mg of amoxicillin (taken three times a day), serum peak levels were between 30.1 and 52.1 μg/mL. It was therefore decided to test the effect of the antibiotics on HepG2 cells in concentrations of 3, 6, 12.5, 25, 50, 100, and 200 μg/mL. The medium was collected for analysis of ApoA‐I concentrations and cells were harvested for analysis of mRNA expression after lysing with TRI reagent. Both medium and lysed cells were snap frozen in liquid nitrogen and stored at −80°C until further analysis.

### 2.3 Quantification of ApoA‐I secretion levels in the culture medium

To investigate ApoA‐I secretion by HepG2 cells, ApoA‐I protein concentrations in culture medium were measured by an enzyme‐linked immunosorbent assay (ELISA) obtained from Mabtech. Direct sandwich ELISA was performed according to the manufacturer's instructions, with slight modifications, blocker BSA 10% (Thermo Fisher Scientific) was added to block buffer and to dilution buffer with a final concentration of 1% and 0.1%, respectively.

### 2.4 Quantification of gene mRNA transcription

To evaluate mRNA expression levels of ApoA‐I, CPT1, KEAP1, and PPARα, total RNA was isolated using TRI reagent, according to the manufacturer's instructions. The RNeasy mini kit (Qiagen, Hilden, Germany) was used for RNA purification. For complementary DNA (cDNA) synthesis, 350 ng of total RNA was reverse‐transcribed using RNAse inhibitor, dNTP's, random hexamers, moloney murine leukemia virus (MMLV) reverse transcriptase, Dithiothreitol (DTT), and 5X first‐strand (FS) buffer (Thermo Fisher Scientific). The resulting cDNA was used for real‐time quantitative polymerase chain reaction using TaqMan Gene Expression assays using cyclophilin A (Hs99999904) as a housekeeping control. To quantify ApoA‐I, KEAP1, PPARα, and CPT1, the following TaqMan Genes Expression assays Hs00163641, Hs00202227, Hs00231882, and Hs00912671 were used. Values are presented as relative gene expressions based on the *C*_t_ values, normalized for the internal control cyclophilin A, and compared with the control conditions.

### 2.5 Luciferase assay

To investigate PPARα transcriptional activity, HepG2 cells were transfected with X‐treme gene 9 DNA transfection reagent (Sigma) and the following plasmids: pcDNA3.1, pcDNA3.1_PPARα, pGL3, and pGL3_PPRE as previously described.^17^ Following transfection and 48 hours SCFA treatment, cells were lysed in luciferase lysis buffer (Promega, Madison, WI), and measured for luciferase activity, reflecting PPARα transactivation, using a GloMax 96 Microplate luminometer, all according to the manufacturer's manual (Promega).

### 2.6 Statistical analysis

All experiments were performed in Duplo and each experiment was repeated three times. To test the dose‐response relationship with the gene of interest, a regression analysis was performed. When the analysis was not‐linear, quadratic polynomial regression was performed. For all statistical analysis, the regression coefficient was considered statistically significant at *P* < .05. Statistical analysis was performed using SPSS v.25 (IBM Corp, Armonk, NY).

## 3 RESULTS

### 3.1 Effects of amoxicillin on ApoA‐I mRNA expression and protein secretion

**Figure 1:**
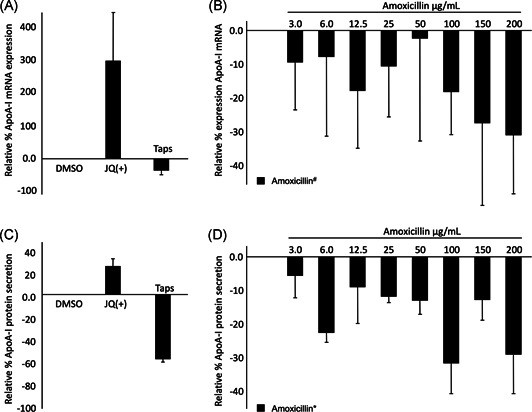
A, Relative ApoA‐I mRNA expression in HepG2 cells treated with JQ1(+) (3 μM) and Taps (0.01 μM). JQ1(+) was used as a positive control for ApoA‐I transcription, whereas Taps was used as a control for reduced ApoA‐I transcription. B, Relative ApoA‐I mRNA expression in HepG2 cells treated with different concentrations of amoxicillin. Increasing amoxicillin concentrations show significant regression coefficient deviated from 0% (*P* < .05) in ApoA‐I mRNA expression. C, Relative ApoA‐I protein secretion by HepG2 cells into the culture medium after treatment with JQ1(+) (3 μM) and Taps (0.01 μM). JQ1(+) was used as a positive control for ApoA‐I protein secretion, whereas Taps was used as a control for reduced ApoA‐I protein secretion. D, Relative ApoA‐I protein secretion by HepG2 cells into the culture medium after treatment with different concentrations of amoxicillin. Increasing amoxicillin concentrations show significant regression coefficient deviated from zero (*P* < .01) in ApoA‐I protein secretion. All results are presented as the mean, while error bars indicate standard deviations. Data were normalized against expression of the control condition, which was arbitrarily set at 0%. Changes are indicated with * when regression coefficient significantly at (*P* < .01) or with ^#^ when regression coefficient significantly at (*P* < .05). ApoA‐I, apolipoprotein‐I; DMSO, dimethyl sulfoxide; mRNA, messenger RNA

Amoxicillin significantly (*P* < .05) and dose‐dependently decreased ApoA‐I mRNA expression up to 30%. Interestingly, the lower ApoA‐I mRNA expression translated into a lower ApoA‐I protein secretion (*P* < .01) into the culture medium, that is ApoA‐I protein concentrations followed mRNA expression. As expected, JQ1(+), a well‐defined BET inhibitor, which was used as a positive control, increased ApoA‐I mRNA expression as well as ApoA‐I protein secretion, while the negative control thapsigargin, an ER‐stress inducer, inhibited both ApoA‐I mRNA expression and protein secretion (Figure 1). Altogether, these findings suggest direct inhibitory effects of amoxicillin on ApoA‐I transcription and secretion.

### 3.2 Effect of SCFA on ApoA‐I mRNA expression and protein secretion

**Figure 2:**
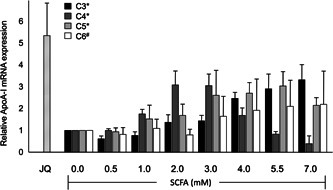
Relative ApoA‐I mRNA expression in HepG2 cells treated with different concentrations of SCFA. Increasing C3, C4, and C5 concentrations show significant regression coefficient deviated from one (*P* < .0001) in ApoA‐I mRNA expression. C6 also show significant regression coefficient deviated from one (*P* < .01) in ApoA‐I mRNA expression. Linear regression for SCFA dose‐response effects was performed except C4, quadratic polynomial regression was performed for C4 dose‐response effects. JQ1(+) (3 μM) was used as a positive control for ApoA‐I transcription. All results are presented as the mean, while error bars indicate standard deviations. Data were normalized against expression of the control condition, which was arbitrarily set at 1. Changes are indicated with a * when regression coefficient significantly at (*P* < .0001) or with a # when regression coefficient significantly at (*P* < .01). ApoA‐I, apolipoprotein‐I; mRNA, messenger RNA; SCFA, short‐chain fatty acids

**Figure 3:**
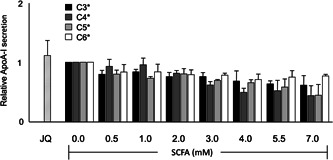
Relative ApoA‐I protein secretion into the culture medium of HepG2 cells after treatment with different concentrations of SCFA. Increasing SCFA concentrations show significant regression coefficient deviated from one (*P* < .05) in ApoA‐I protein secretion. JQ1(+) (3 μM) was used as a positive control for ApoA‐I protein secretion. All results are presented as the mean, while error bars indicate standard deviations. Data were normalized against expression of the control condition, which was arbitrarily set at 1. Changes are indicated with a * when regression coefficient significantly at (*P* < .05). ApoA‐I, apolipoprotein‐I; SCFA, short‐chain fatty acids

The positive control JQ1(+) strongly increased ApoA‐I mRNA expression in all experiments with SCFA (Figure 2). This indicated that the cells were responsive and allows further analysis with SCFA. ApoA‐I mRNA expression dose‐dependently increased after C3 treatment (*P* < .0001), with a maximum of a 3.2‐fold increase at 7 mM. Treatment with C4 first dose‐dependently increased ApoA‐I mRNA expression which resulted in a three‐fold increase at 3 mM followed by a dose‐dependent decrease at higher concentrations down to ±50% of basal ApoA‐I mRNA expression at 7 mM (*P* < .0001). C5 dose‐dependently and significantly (*P* < .0001) increased ApoA‐I mRNA expression up to a three‐fold increase at 5.5 mM. C6 gradually increased ApoA‐I mRNA expression with a maximum of two‐fold at 7 mM (*P* < .01; Figure 2). Although ApoA‐I mRNA expression was elevated with all types of SCFA, except higher C4 doses, we observed for all SCFA a dose‐dependent decrease in ApoA‐I protein concentrations in the culture medium of the HepG2 cells (*P* < 0.05; Figure 3). In contrast, the positive control JQ1(+) not only elevated ApoA‐I mRNA expression but also increased ApoA‐I protein secretion in the culture medium (Figure 3).

### 3.3 Effect of SCFA on KEAP1, CPT1, and PPARα mRNA expression

To evaluate potential underlying mechanisms, changes in activities on the BET and PPARα pathways were evaluated. In general, BET inhibition is related to a decreased expression of the BET target gene KEAP1 and PPARα transactivation via increased expression of the PPARα target genes CPT1 and PPARα itself.

**Figure 4:**
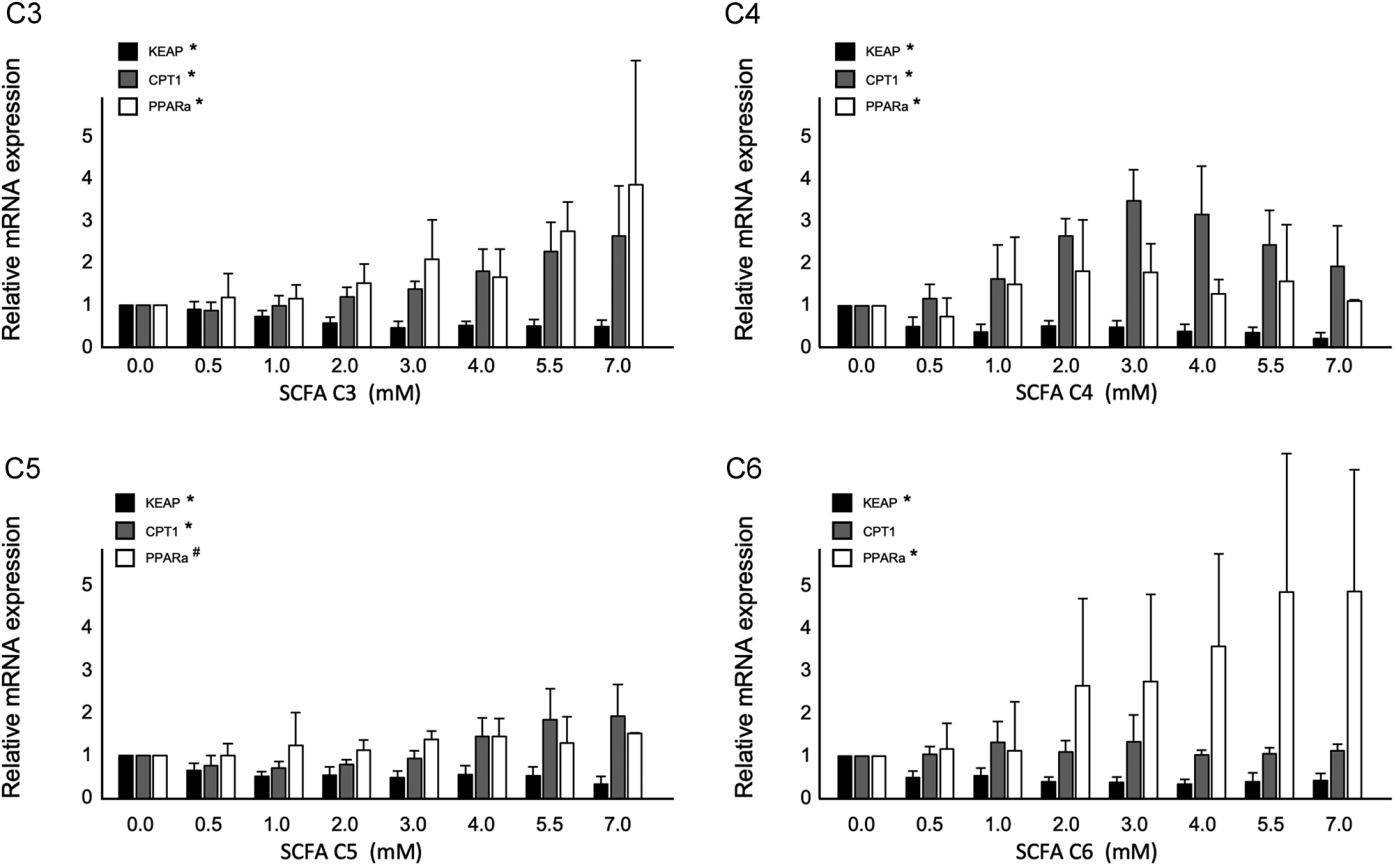
Relative KEAP1, CPT1, and PPARα mRNA expression in HepG2 cells treated with different concentrations of SCFA. C3 different concentrations show significant regression coefficient deviated from one (*P* < .0001) in KEAP1, CPT1, and PPARα mRNA expression. C4 different concentrations show significant regression coefficient deviated from one (*P* < .0001) in KEAP1 and CPT1 and PPARα mRNA expression. C5 different concentrations show significant regression coefficient deviated from one (*P* < .0001) in KEAP1 and CPT1, (*P* < .01) in PPARα mRNA expression. C6 different concentrations show significant regression coefficient deviated from one (*P* < .0001) in KEAP1 and PPARα mRNA expression. Linear regression for SCFA dose‐response effects was performed except C4, quadratic polynomial regression was performed for C4 dose‐response effects. All results are presented as the mean, while error bars indicate standard deviations. Data were normalized against expression of the control condition, which was arbitrarily set at 1. Changes are indicated with a * when regression coefficient significantly at (*P* < .0001) or with a # when regression coefficient significantly at (*P* < .01). mRNA, messenger RNA; SCFA, short‐chain fatty acids

In general, SCFA treatment significantly and dose‐dependently decreased KEAP1 gene expression (*P* < .0001). In more detail, C3 decreased KEAP1 gene expression maximally by 50% at 3 mM, whereas C4 lowered KEAP1 expression even up to 80% at 7 mM. Moreover, KEAP1 expression decreased by 60% and 50% after exposure to 3 mM C5 and C6, respectively. Interestingly, KEAP1 was downregulated while ApoA‐I mRNA expressions was increased for all SCFA, suggesting that BET inhibition could be related to the observed increases in ApoA‐I mRNA levels (Figure 4).

Moreover, CPT1 mRNA expression dose‐dependently and significantly increased after SCFA treatment. More specifically, C3 significantly (*P* < .0001) increased CPT1 mRNA expression with a maximum of a 2.7‐fold at 7 mM. C4 was the strongest activator, that is a 3.6‐fold CPT1 expression at 3 mM. In line with ApoA‐I, this C4‐induced elevation in CPT1 expression was followed by gradual reductions at higher C4 doses (*P* < .0001). C5 significantly (*P* < 0.001) increased CPT1 gene expression to 1.9‐fold at 7 mM, whereas C6 was the weakest activator, that is only a slight but not significant 1.2‐fold increase at 3 mM was observed (Figure 4).

In line with CPT1, all SCFA elevated PPARα gene expression, although the effects of C4 fluctuated. Both C3 and C6 significantly increased PPARα expression to 4.0‐fold and 4.4‐fold, respectively (*P* < .0001). Like for ApoA‐I and CPT1, C4 increased PPARα expression 1.9‐fold with the most pronounced effect at 3 mM. At higher doses, a reduction of PPARα expression was observed. C5 significantly (*P* < .01) increased PPARα expressions to 1.8‐fold at 7 mM (Figure 4).

**Figure 5:**
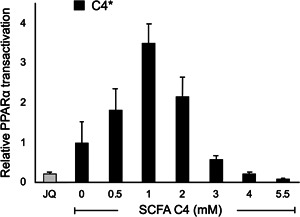
Relative PPARα transactivation in transfected HepG2 cells treated with different concentrations of C4. Increasing C4 concentrations show significant regression coefficient deviated from one (*P* < .0001) in PPARα transactivation. All results are presented as the mean, while error bars indicate standard deviations. Data were normalized against expression of the control condition, which was arbitrarily set at 1. Changes are indicated with a * when regression coefficient significantly at (*P* < .0001)

To evaluate whether the effects of SCFA on CPT1 and PPARα mRNA expressions could be explained via PPARα activation, we evaluated changes in PPARα transcriptional activity with C4 as a model compound for all SCFA tested, in transfected HepG2 cells using a PPRE luciferase reporter system. In line with ApoA‐I, CPT1, and PPARα mRNA expressions, C4 significantly increased PPARα transactivation, followed by a gradual dose‐dependent decrease resulting in an inhibition of 90% of basal PPARα transactivation at 5.5 mM (*P* < 0.0001). Interestingly, JQ1(+) markedly inhibited PPARα transactivation by 70% (Figure 5).

Overall, for all SCFA the patterns between CPT1 as well as PPARα and ApoA‐I mRNA expressions and PPARα transactivation were comparable suggesting that PPAR activation might relate to the observed elevation in ApoA‐I mRNA levels.

## 4 DISCUSSION

We here report a series of in vitro experiments in HepG2 cells supporting our hypothesis that antibiotics could have direct and indirect effects on hepatic ApoA‐I transcription and secretion. First, we showed that amoxicillin lowered ApoA‐I transcription and secretion by HepG2 cells, whereas all SCFA tested, which are metabolites produced by microbiota, clearly increased ApoA‐I transcription, except for C4 at higher concentrations. This suggests that increasing the flux of SCFA from the portal vein towards the liver might translate into an increased hepatic ApoA‐I transcription. Mechanistically, we showed that SCFA‐induced BET inhibition as well as PPARα activation suggesting that these two pathways are potentially involved.

Recently, we observed in healthy human volunteers that amoxicillin, a broad‐spectrum antibiotic, significantly lowered serum HDL‐C concentrations.^2^ Amoxicillin is a member of β‐lactam antibiotics, which negatively affects bacterial growth by binding to a primary receptor, called the penicillin‐binding protein, on the bacterial membrane.^21^, ^22^ As a consequence, bacteria lose their control over autolytic enzymes leading to apoptosis.^23^ As such, amoxicillin has a major impact on microbiota composition and given the enormous number of observations regarding the role of microbiota on human metabolism, antibiotics could theoretically affect numerous processes.^24^ Besides a direct effect of amoxicillin, our findings also suggest that changing microbiota composition which theoretically also changes SCFA production, impact hepatic ApoA‐I expression. In other words, besides the direct gene regulating effect, it is well possible that the observed effects of amoxicillin treatment in humans on HDL cholesterol concentrations are indirect, that is, mediated by altering the composition of the microbiota, which are responsible for the production of SCFA.^1^

ApoA‐I mRNA expression was stimulated by C3, C4, C5, and C6 treatments. At the same time, BET was inhibited and PPARα transactivated, suggesting that both BET inhibition, as well as PPARα activation, are involved in the effect of SCFA on ApoA‐I expression. Interestingly, PPARα transactivation closely followed the same pattern, though not fully, which suggests that additional factors could be involved. These factors might include large families of coactivators and corepressors molecules, which together determine the net outcome of PPARα mediated effects.^25^ PPARα can be activated by recruitment of coactivators such as CBP and SRC1, but can be inhibited by binding of corepressors such as SMRT and NCoR.^26^ On the other hand, increased ApoA‐I expression with the BET inhibitor JQ1(+) was observed, even though JQ1(+) failed to induce PPARα transactivation. This indicates that ApoA‐I expression could also increase due to other mechanisms besides PPARα transactivation, for instance via BET inhibition. This is also in agreement with our previous research in which we showed a clear role for BET inhibition, for example via exposure to JQ1(+), on ApoA‐I expression.^10^ JQ1(+) binds to acetylated lysine binding sites within BET bromodomains, leading to BET proteins dissociation from chromatin, amongst others translating into elevated ApoA‐I mRNA expression in HepG2 cells.^27^ In addition, JQ1(+) also inhibited PPARα transactivation as PPARα and CTP1 expressions were both downregulated. This means that the net results of BET inhibitors on ApoA‐I are actually very strong, since they also need to overcome the effect of a lower PPARα activation.

A puzzling question that remains is why after exposure to the SCFA, ApoA‐I concentrations in culture medium were in contrast to the elevated ApoA‐I mRNA levels, significantly reduced. The lower secretion of ApoA‐I into culture medium suggests that the higher level of ApoA‐I mRNA is not translated or ApoA‐I protein is produced but possibly remains trapped within the hepatocytes. Furthermore, it is also possible that ApoA‐I was internalized and recycled back to the cell surface, which might be the reason behind the reduced level of protein secreted.^28^ Moreover, the incubation period 48 hours seems suitable for analyzing changes in ApoA‐I mRNA expression, but for the changes in protein secretion, this time frame could be relatively short. A study by Hahn and Goldberg^29^ has shown a significant effect on ApoA‐I protein secretion only after 3 days of incubation. However, it should be mentioned that our positive control JQ1(+) did elevate ApoA‐I protein secretion after 48 hours.

We observed some variation in SCFA ability to increase ApoA‐I expression. This variation might be due to the differences in the binding strength of the SCFA to PPARα or BET. As mentioned before, several of coactivators and corepressors molecules mediate PPARα effects,^25^ SCFA (eg, C4 at higher doses) may activate these corepressor domains instead of activating the coactivator domains of PPARα, which can explain the inhibitory effects of C4 higher doses on PPARα transactivation and ApoA‐I mRNA expression as well.

Of all SCFA evaluated, C4 was at lower doses the most promising SCFA regarding elevating ApoA‐I expression. Therefore, it might be interesting to evaluate the effects of C4 on ApoA‐I production in future human intervention studies. Previous study has demonstrated that specific fibers such as resistant starch increased C4 formation by the microbiota.^30^ Biscuits rich in resistant starch type 3 improved glucose and lipid profiles in diabetic mice.^31^ Furthermore, a positive correlation between starch‐rich food consumption and increased number of HDL particles was observed, this correlation resulted in lipid profile improvement.^32^

In summary, we have shown that amoxicillin treatment has either direct effects on lowering ApoA‐I transcription and secretion or indirect effects via modified SCFA concentrations, because SCFA were found to stimulate hepatic ApoA‐I expression. Effects of all four SCFA tested on ApoA‐I mRNA expression showed a dose‐dependent increase, except for C4 higher doses. Finally, based on the findings regarding KEAP1, CPT1, and PPARα gene expression as well as PPAR transactivation, it is tempting to suggest that both BET inhibition and PPARα activation are potential mechanisms behind the observed SCFA‐induced effects on ApoA‐I expression. A more detailed understanding of these mechanisms would add to new insights for approaches in elevating HDL levels to prevent and treat atherosclerosis and CVD.

## CONFLICT OF INTERESTS

The authors declare that there is no conflict of interests.

## AUTHOR CONTRIBUTIONS

All authors substantially contributed to conception and design, acquisition of data, or analysis and interpretation of data. All authors substantially contributed to drafting the article or revising it critically for important intellectual content. All authors substantially contributed to final approval of the version to be published.
